# Risk Factors for Postischemic Stroke Epilepsy in Young Adults: A Nationwide Population-Based Study in Taiwan

**DOI:** 10.3389/fneur.2022.880661

**Published:** 2022-05-20

**Authors:** Phuong Thao Do, Li-Ying Chen, Lung Chan, Chaur-Jong Hu, Li-Nien Chien

**Affiliations:** ^1^International Ph.D. Program for Cell Therapy and Regeneration Medicine, College of Medicine, Taipei Medical University, Taipei, Taiwan; ^2^Department of Pediatrics, Hanoi Medical University, Hanoi, Vietnam; ^3^Health Data Analytics and Statistics Center, Office of Data Science, Taipei Medical University, Taipei, Taiwan; ^4^Department of Neurology and Stroke Center, Shuang Ho Hospital, Taipei Medical University, New Taipei City, Taiwan; ^5^PhD Program in Medical Neuroscience, College of Medical Science and Technology, Taipei Medical University, Taipei, Taiwan; ^6^Taipei Neuroscience Institute, Taipei Medical University, Taipei, Taiwan; ^7^Department of Neurology, School of Medicine, College of Medicine, Taipei Medical University, Taipei, Taiwan; ^8^School of Health Care Administration, College of Management, Taipei Medical University, Taipei, Taiwan

**Keywords:** ischemic stroke, poststroke epilepsy, epilepsy, young adults, risk factors, stroke severity, comorbidities, unhealthy lifestyles

## Abstract

**Background:**

The incidence of ischemic stroke has been increasing in the young population over the past 20 years. Poststroke epilepsy (PSE) is a common complication after stroke. However, few population-based studies with sufficient follow-up have investigated factors associated with PSE, especially factors related to comorbidities and unhealthy lifestyles in the modern young population. Accordingly, this study aimed to determine the long-term incidence and these risk factors for PSE young adults.

**Methods:**

This cohort study was conducted using data from the Taiwan National Health Insurance Research Database (NHIRD) from 2002 to 2018. All patients aged between 19 and 44 years and diagnosed with ischemic stroke from 2002 to 2015 were retrospectively enrolled with a follow-up of at least 3 years. Multivariable Cox regression models were performed to identify predictors of PSE, including patients' demographics, baseline conditions, stroke severity, etiologies, comorbidities, and unhealthy behaviors.

**Results:**

Among 6,512 ischemic stroke patients, 402 cases (6.2%) developed PSE who were with a mean follow-up period of 8.3 years (SD = 4.3 years). During the overall follow-up, stroke severity and manifestations were associated with PSE, including National Institutes of Health Stroke Scale (NIHSS) score ≥10 (aHR, 1.98; 95% CI, 1.50–2.61), seizure at first stroke admission [adjusted hazard ratio (aHR), 57.39; 95% confidence interval (CI), 43.02–76.55], length of hospital stay ≥14 days (aHR, 1.60; 95% CI, 1.26–2.02), recurrent stroke (aHR, 2.32; 95% CI, 1.85–2.90), aphasia (aHR, 1.77; 95% CI, 1.20–2.60), and malignancy (aHR, 2.05; 95% CI, 1.30–3.24). Furthermore, stroke patients with drug abuse were 2.90 times more likely to develop PSE than those without (aHR, 2.90; 95% CI, 1.53–5.50). By contrast, statin use (aHR, 0.62; 95% CI, 0.48–0.80) was associated with a lower risk of PSE. The risk factors at 1-year and 5-year PSE were similar to that of an overall follow-up.

**Conclusions:**

Stroke severity, aphasia, malignancy, and drug abuse were associated increased risk of PSE and statin use may protect against PSE in young adults. Reducing the severity of stroke, statin use and controlling unhealthy behaviors might be able to decrease the development of PSE. Since PSE is associated with poor outcomes, early identification or intervention of PSE based on the risk factors might reduce the harmful effects of PSE.

## Introduction

Cerebrovascular diseases are the leading cause of mortality and long-term disability worldwide (1, 2). However, the global incidence of stroke in the younger population has been increasing over the past 20 years (1, 3–6). From 1995 to 2012, the National Inpatient Sample in the United States reported an almost doubled rate of ischemic stroke hospitalization in patients aged 18–44 years while remaining stable for intracerebral hemorrhage and subarachnoid hemorrhage (5). Furthermore, young adult stroke patients may have long-term complications, including functional disability, depression, cognitive impairment, and unemployment (3, 4, 7, 8). A common complication after cerebral infarction in young adults is poststroke epilepsy (PSE) (9). Depending on the subtype of stroke and the follow-up time, 2.7% to 12.7% of patients with stroke developed PSE (10–15). The risk of epilepsy following ischemic stroke is lower than that after hemorrhagic stroke and has been reported to be between 2.7 and 6.6% (10, 12, 13). However, the number of PSE patients is persistently high because of the higher prevalence of ischemia than that of hemorrhage in young adults (9). Moreover, PSE is associated with increased short-term and long-term mortality and impaired quality of life (10, 16). PSE patients also had poorer functional outcomes measured by both the modified ranking scale (mRS) and the Instrumental Activities of Daily Living (iADL) scale than those without PSE (27.5% vs. 9.8% for mRS > 2; 27.8% vs. 12.6% for iADL < 8) (11).

Early detection of risk factors and preventive interventions might reduce the occurrence of PSE. Therefore, studies have been conducted to identify the risk factors for PSE. Prior studies showed poststroke symptomatic seizures were an important risk factor for PSE (17). Approximately 30% of stroke patients with acute symptomatic seizures develop PSE (17). In addition to seizure, the severity of stroke (NIHSS score, coma, and length of hospitalization) (9, 14, 18), localized characteristics of lesions (focal neurological signs, cortical involvement, aphasia and visual field defect) (10, 14, 19–21), and stroke recurrence (15) have been reported to be major predictive factors for PSE. Furthermore, the age rejuvenation trend of ischemic stroke might be associated with the prevalence of some traditional stroke risk factors, including modifiable risk factors (obesity, hyperglycemia, hyperlipidemia, and hypertension) and behavioral risk factors (smoking, heavy drinking, drug abuse, and sedentary lifestyle). These factors are increasing among young patients hospitalized for cerebral infarction (3, 5, 22). Nevertheless, the evidence for these comorbidities influencing PSE risk is inconsistent. Evidence demonstrated an increased risk of PSE in stroke patients with hypertension, hyperlipidemia, or diabetes (23). By contrast, no correlation was found in another study (24). Some unhealthy behaviors such as smoking, drinking, and drug abuse have emerged as risk factors for the onset and recurrence of stroke in young adults in modern life (25, 26). Hitherto, these are still unclear prognostic factors for PSE (27, 28). Notably, illicit and recreational drug use (cocaine, cannabis, and opioids) has increased over the past decade and is more frequently associated with stroke in the young population (29). To the best of our knowledge, there is no research to discuss the potential association between these unhealthy lifestyle risk factors and PSE in young adults, especially illegal drug abuse.

A better definition of the factors associated with PSE could help target populations that would benefit from therapies to reduce epileptogenesis. Although risk factors of PSE in young adults have been reported but limitations exist, such as small sample size, short follow-up period, and heterogeneous stroke subtypes (transient ischemic attack, ischemic stroke, and intracerebral hemorrhage) (9, 27, 28). A population-based study would enable a comprehensive and accurate determination of the long-term incidence and risk factors for PSE. In particular, we focus on unclear risk factors for ischemic stroke that especially appear in young populations, including comorbidities and unhealthy lifestyles. Therefore, we conducted this retrospective cohort study to investigate the long-term incidence and predictive factors of PSE in young adults based on data from the Taiwan National Health Insurance Research Database (NHIRD).

## Materials and Methods

### Study Population and Design

In this retrospective cohort study, patients were selected from the NHIRD, a population-based database of claims data from the National Health Insurance (NHI) program of Taiwan. Almost the entire population of Taiwan is covered by the NHI program; thus, the database is comprehensive. The data files in this study are maintained by the Health and Welfare Data Science Center of the Ministry of Health and Welfare. All patients aged 19–44 years with ischemic stroke from 2002 to 2015 were enrolled in this study with follow-up of at least 3 years. Admissions for ischemic stroke were first identified using *International Classification of Diseases, Ninth Revision, Clinical Modification* (ICD-9-CM) codes for ischemic stroke with any discharge coding as follows: 433 (occlusion and stenosis of precerebral arteries), 434 (occlusion of cerebral arteries), 436 (acute, but ill-defined, cerebrovascular disease), 437 (other and ill-defined cerebral vascular disease). In all cases, diagnosis was confirmed by brain computed tomography (CT) (payment code of 33067B, 33068B, and 33069B) or magnetic resonance imaging (MRI) (33084A, 33084B, 33085A, and 33085B). We treated the first date of ischemic stroke was the index date.

**Figure 1:**
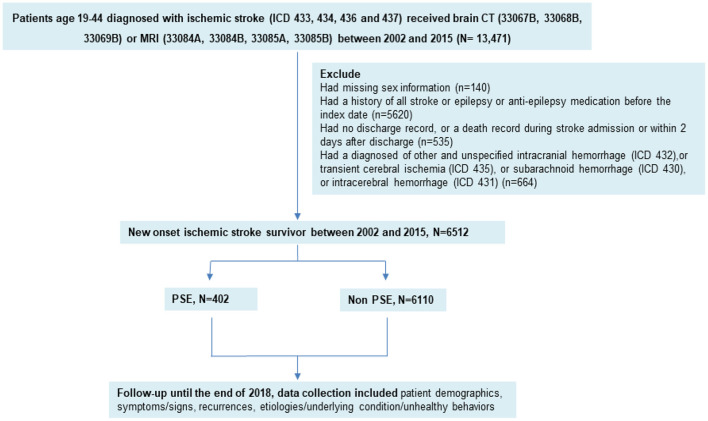
Flowchart of patient recruitment.

The exclusion criteria were as follows: patients had been diagnosed with other and unspecified intracranial hemorrhage (ICD-9 code 432), transient cerebral ischemia (ICD-9 code 435), subarachnoid hemorrhage (ICD-9 code 430), intracerebral hemorrhage (ICD-9 code 431), history of ischemic stroke, or epilepsy (ICD-9 code 435); those taking antiepileptic medication before the index date; and those with a lack of information (such as no discharge record or missing sex information) or a death record during stroke admission or within 2 days after discharge (Figure 1).

### Outcome Measurement

PSE was defined if patients had a diagnosis of epilepsy (ICD-9 code 345, ICD-10 code G40 after 2016) and recurrent seizures (ICD-9 codes 780.3X and 780.39, ICD-10 codes G40) and a pharmacy claim for antiepileptic drugs. Each patient was followed from the discharge date of the index stroke to 1) the date of first PSE occurrence, 2) the date of death or 3) the date of December 31th, 2018 which was the last date of follow-up period.

The data collection included patient demographics (gender, age), symptoms/signs (NIHSS score, symptoms during first stroke admission, length of hospital stay), stroke recurrence during follow ups, etiologies, underlying condition, and some behavioral risk factors. NIHSS score was measured using the treatments and procedures at admission, including nasogastric intubation, ICU stay, urinary catheterization, airway suctioning, osmotherapy, general ward stays, and bacterial sensitivity test to create a proxy indictor represented NHISS score (30). The etiologies and comorbidities of ischemic stroke patients were defined based on ICD-9 codes described in the Supplementary Material (Supplementary Table 2). Comorbidities were included if patients had at least two relevant clinic visits 1 year prior to admission for stroke.

### Statistical Analysis

Baseline characteristics, clinical presentations, etiologies, underlying conditions, and behavioral risk factors of stroke patients with and without PSE were compared using the chi-square test, Fisher's exact test, and *t*-test. The cumulative risk of PSE was estimated through Kaplan–Meier analysis. Factors for PSE over time were evaluated using multivariate Cox proportional hazard models along with hazard ratios (HRs) and 95% confidence intervals (CIs). Stroke recurrence was treated as a time-dependent variable into the models. A two-sided *p*-value of < 0.05 was considered statistically significant.

## Results

### Patients' Baseline and Clinical Characteristics

The sample included 13,471 patients from the NHIRD aged 19–44 years and diagnosed with ischemic stroke from 2002 to 2015. A total of 6512 young adults (64.5% men, aged 37.19 ± 6.14 years with median age =39 years) at the first episode of ischemic stroke were enrolled into the study. During a mean follow-up period of 8.3 years (SD = 4.3 years), 402 (6.2%) developed PSE. PSE patients were remarkably younger (36.20 ± 6.76 years with a median age = 38 years) than stroke patients without PSE (37.25 ± 6.09 years with a median age = 39 years) (*p* < 0.001) while no different in gender (*p* = 0.754). Baseline characteristics are presented in Table 1.

**Table 1:** Baseline characteristics and clinical presentations of young adults with poststroke epilepsy at first stroke admission.

	**Poststroke epilepsy**						
	**Total**, ***N*** **=** **6512**		**Present**, ***N*** **=** **402**		**Absent**, ***N*** **=** **6110**		**p-value**
	***n***	**%**	**n**	**%**	**n**	**%**	
Seizure at first stroke admission	96	1.5	87	21.6	9	0.1	<0.001
Male	4197	64.5	262	65.2	3935	64.4	0.754
Age (mean (SD), median (years))	37.19 (6.14),39		36.20 (6.76),38	37.25 (6.09),39	0.001		
Admission NIHSS score							
0–4	4888	75.1	217	54.0	4671	76.4	<0.001
5–9	1009	15.5	77	19.2	932	15.3	
≥10	615	9.4	108	26.9	507	8.3	
Intravenous thrombolysis, yes	198	3.0	14	3.5	184	3.0	0.594
Symptoms during first stroke admission							
Focal neurological signs	1005	15.4	74	18.4	931	15.2	0.088
Hemiparesis	832	12.8	58	14.4	774	12.7	0.306
Cranial nerve palsy	125	1.9	6	1.5	119	1.9	0.520
Aphasia	214	3.3	30	7.5	184	3.0	<0.001
Vertigo, dizziness	509	7.8	20	5.0	489	8.0	0.029
Coma, Consciousness disturbance	24	0.4	4	1.0	20	0.3	0.057
Headache	568	8.7	45	11.2	523	8.6	0.070
Sleep disorder	108	1.7	9	2.2	99	1.6	0.347
Length of hospital stay, mean (SD), median	10.25 (10.29),7		14.30 (15.05),9		9.98 (9.84),7		<0.001
Recurrent stroke readmission	1228	18.9	127	31.6	1101	18.0	<0.001

Seizure at first stroke admission (*n* = 96) occurred at a cumulative rate of 1.5%. The frequency of seizure at first stroke admission was 21.6% and 0.1% for patients with and without PSE, respectively [adjusted HR (aHR), 57.39; 95% CI, 43.02–76.55]. Poststroke seizures can occur soon after the onset of ischemia or can be delayed. The occurrence of seizure within the 1st year after the index stroke was 55.7% (*n* = 224), decreased to 13.4% (*n* = 54) in the second year, and was 7.0% (*n* = 28), 5.5% (*n* = 22), and 3.0% (*n* = 12) in the third, fourth, and fifth years, respectively (data not show).

In addition, the clinical severity of stroke patients assessed based on the NIHSS score and length of hospital stay was proportional to the rate of PSE progression. In the PSE group, 19.2% and 26.9% of patients had NIHSS scores of 5–9 and ≥10, respectively, which were markedly higher than those in the non-PSE group (15.3% and 8.3%), respectively (*p* < 0.001). A higher mean length of stay was also observed in patients with PSE (14.30 ± 15.5 days, median = 9 days) than in those without PSE (9.98 ± 9.84 days, median = 7 days) (*p* < 0.001). Furthermore, the rate of recurrent stroke in PSE patients (31.6%) was significantly higher than that in patients without PSE (18.0%) (*p* < 0.001).

Most clinical symptoms at first stroke admission were not significantly different for patients with and without PSE, including focal neurological signs (*p* = 0.088), hemiparesis (*p* = 0.306), cranial nerve palsy (*p* = 0.520), consciousness disturbance (*p* = 0.057), headache (*p* = 0.07), and sleep disorder (*p* = 0.347). However, 30 (7.5%), and 20 (5.0%) PSE stroke patients had aphasia, and vertigo/dizziness, respectively; these rates were elevated compared with those in stroke patients without PSE (*p* < 0.001, *p* = 0.029, respectively).

### Etiology, Underlying Conditions, and Behavioral Risk Factors for Ischemic Stroke and PSE

We evaluated the leading causes of ischemic stroke in young adults in Table 2. Among the common underlying conditions of stroke, only malignancy was significantly associated with PSE progression (*p* = 0.009) (Shown in Table 3). Notably, the prevalence of hyperlipidemia, and statin therapy in PSE patients was significantly lower than in patients without PSE (*p* < 0.001). Furthermore, behavioral risk factors such as drug abuse, smoking, and drinking were associated with an increased risk of PSE (*p* < 0.001; *p* = 0.003; and *p* = 0.012, respectively).

**Table 2:** Etiology of ischemic stroke and PSE development.

	**Poststroke epilepsy**						
	**Total**, ***N*** **=** **6512**		**Present**, ***N*** **=** **402**		**Absent**, ***N*** **=** **6110**		**p value**
	**n**	**%**	**n**	**%**	**n**	**%**	
**Artery atherosclerosis**	5442	83.6	333	82.8	5109	83.6	0.682
**Non-atherosclerotic angiopathies**							
Systemic vasculitis, systemic lupus	269	4.1	17	4.2	252	4.1	0.919
Cerebral aortic aneurysm	201	3.1	14	3.7	187	3.1	0.636
Moya Moya	62	1.0	5	1.7	57	0.9	0.433
Ill-defined cerebrovascular disease, small vessel disease	75	1.2	5	1.2	70	1.2	0.808
**Cardio embolism**							
Congenital complex heart syndromes	77	1.2	7	1.7	1.1	70	0.333
Atrial fibrillation, other arrhythmias	209	3.2	15	3.7	3.2	194	0.540
Heart valve disease, endocarditis	27	0.4	0	0.0	0.4	27	0.409
Coronary artery disease, myocardial infarction	296	4.5	13 3.2	4.6	283	0.192
Hypertrophic cardiomyopathy, heart failure	278	4.3	23	5.7	255	4.2	0.137
**Congenital metabolic disorders**	63	1.0	-	-	-	-	0.184
**Coagulopathy and hematology**	37	0.6	-	-	-	-	1.000
**Systemic infections**	38	0.6	-	-	-	-	0.729

*Dash indicates <3 patients*.

**Table 3:** Stroke risk factors and underlying conditions in PSE patients.

	**Poststroke epilepsy**						
	**Total**, ***N*** **=** **6512**		**Present**, ***N*** **=** **402**		**Absent**, ***N*** **=** **6110**		**p-value**
	**n**	**%**	**n**	**%**	**n**	**%**	
**Comorbidities**							
Hyperlipidemia	2116	32.5	79	19.7	2037	33.3	<0.001
Chronic head and neck disorders	933	14.3	61	15.2	872	14.3	0.617
Diabetes mellitus, type 2	1060	16.3	56	13.9	1004	16.4	0.188
Diabetes mellitus, type 1	1144	17.6	61	15.2	1083 17.7	0.193	
Migraine	157	2.4	9	2.2	148	2.	0.816
Malignancy	187	2.9	20	5.0	167	2.7	0.009
Moderate or severe liver disease	19	0.3	3	0.7	16	0.3	0.109
Nephrotic syndrome	37	0.6	5	1.2	32	0.5	0.075
Renal disease	291	4.5	13	3.2	278	4.5	0.216
Rheumatoid arthritis, collagen vascular disease	273	4.2	16	4.0	257	4.2	0.827
**Behavioral risk factors**							
Drug abuse	36	0.6	10	2.5	26	0.4	<0.001
Gravidity or postpartum period	154	2.4	9	2.2	145	2.4	0.864
Tobacco smoking	78	1.2	12	3.0	66	1.1	0.003
Regular drinking/heavy drinking	167	2.6	18	4.5	149	2.4	0.012
**Statin treatment**	1930	29.6	77	19.2	1853	30.3	<0.001

### Predictive Factors for PSE in Stroke Patients Aged 19–44 Years

Overall, patients with a high NIHSS score at admission (aHR = 1.40 for NIHSS score 5–9; aHR = 1.98 for NIHSS score ≥10), aphasia (aHR = 1.77), length of hospital stay ≥14 days (aHR = 1.60), recurrent stroke (aHR = 2.32), malignancy (aHR = 2.05), and drug abuse (HR = 2.90) had an increased risk of PSE in multivariate Cox proportional hazard models (Table 4). Hyperlipidemia was associated with PSE risk reductions by 33% 1 years following stroke onset. Notably, statin treatment diminished the risk of progression to PSE by 54% and 44% at 1 and 5 years after stroke, respectively.

**Table 4:** Multivariate Cox regression analysis of risk factors for PSE in stroke patients aged 19–44 years.

	**Timing of PSE occurrence**								
	**All**			**1 year**			**5 years**		
	**aHR**	**95% CI**	***p***	**aHR**	**95% CI**	***p***	**aHR**	**95% CI**	***p***
Seizure									
Yes	57.39	43.02–76.55	<0.001	56.47	40.74–78.27	<0.001	54.76	40.74–73.61	<0.001
No	1.00	Ref.		1.00	Ref.		1.00	Ref.	
Age (years)									
19-34	1.00	Ref.	0.557	1.00	Ref.	0.840	1.00	Ref.	<0.619
35-44	1.07	0.86–1.34		1.03	0.77–1.38		1.06	0.84–1.35	
NIHSS score									
0–4	1.00	Ref.		1.00	Ref.		1.00	Ref.	
5–9	1.40	1.07–1.84	0.015	1.50	1.05–2.15	0.027	1.47	1.09–1.97	0.011
≥10	1.98	1.50–2.61	<0.001	1.97	1.36–2.84	<0.001	2.13	1.58–2.88	<0.001
Aphasia									
Yes	1.77	1.20–2.60	0.004	1.93	1.17–3.17	0.010	1.866	1.23–2.80	0.003
No	1.00	Ref.		1.00	Ref.		1.00	Ref.	
Vertigo/dizziness									
Yes	0.70	0.44–1.09	0.117	0.61	0.31–1.20	0.153	0.79	0.49–1.27	0.332
No	1.00	Ref.		1.00	Ref.		1.00	Ref.	
Length of hospital stay									
<14 days	1.00	Ref.		1.00	Ref.		1.00	Ref.	
≥14 days	1.60	1.26–2.02	<0.001	1.57	1.15–2.16	0.005	1.67	1.29–2.16	<0.001
Recurrent stroke									
Yes	2.32	1.85–2.90	<0.001	1.32	0.93–1.87	0.116	1.82	1.41–2.36	<0.001
No	1.00	Ref.		1.00	Ref.		1.00	Ref.	
Hyperlipidemia									
Yes	0.82	0.65–1.04	0.098	0.67	0.48–0.94	0.020	0.73	0.57–0.95	0.020
No	1.00	Ref.		1.00	Ref.		1.00	Ref.	
Statin treatment									
Yes	0.62	0.48–0.80	<0.001	0.46	0.31–0.68	<0.001	0.56	0.41–0.74	<0.001
No	1.00	Ref.		1.00	Ref.		1.00	Ref.	
Malignancies									
Yes	2.05	1.30–3.24	0.002	1.55	0.81–2.96	0.184	1.82	1.09–3.03	0.022
No	1.00	Ref.		1.00	Ref.		1.00	Ref.	
Drug abuse									
Yes	2.90	1.53–5.50	0.001	2.73	1.18–6.30	0.018	2.61	1.28–5.33	0.009
No	1.00	Ref.		1.00	Ref.		1.00	Ref.	
Heavy drinking									
Yes	1.17	0.52–2.63	0.708	1.33	0.49–3.60	0.578	1.35	0.60–3.06	0.465
No	1.00	Ref.		1.00	Ref.		1.00	Ref.	
Tobacco smoking									
Yes	0.88	0.28–2.78	0.824	0.88	0.21–3.65	0.857	0.59	0.17–2.05	0.407
No	1.00	Ref.		1.00	Ref.		1.00	Ref.	

## Discussion

Several predictive factors for PSE were determined through the 16-year follow-up among patients aged 19–44 years after ischemic stroke, including seizure at first admission of stroke, aphasia, severity of stroke (high NIHSS score and prolonged hospital stay), recurrent stroke, and drug abuse. The incidence of PSE following ischemic stroke was 6.2%, consistent with the rate of 2.7%−6.6% in previous studies (10, 12, 13, 24). Our results also revealed that PSE more frequently occurred in younger patients (*p* = 0.001). Patient age is still a controversial prognostic factor for PSE. Numerous studies have demonstrated that younger age is a critical risk factor for PSE (9, 14, 28), whereas other studies reported no association between age and PSE (15, 27). The heterogeneity in the age of hospitalized patients and the subtype of stroke may cause these conflicting results.

Because of distinct characteristics and mechanisms of seizure, most studies set a 7-day interval to categorize early- and late-onset poststroke seizures (9, 11, 17, 19, 27). However, this study was unable to identify the exact timing of the first seizure after stroke onset due to the limitation of using the Taiwan NHI research database. We, alternatively, used seizure in the first episode of hospitalized stroke as the proxy variable, which accounts for 1.5%, were significantly more present in PSE patients (*p* < 0.001). Thus, we concluded that seizure at first stroke admission is a critical predictor of future PSE. This finding is consistent with previous studies, which indicated that both acute symptomatic seizures and late seizures were associated with a higher risk of developing PSE in the young population (19, 27). The risk of epilepsy following stroke is the highest in the first 2 years, but it continues to be high even 10 years after stroke (14, 15, 21). Although seizure incidence after ischemic stroke peaks within 1 year after cerebral insult (55.7%), the occurrence of seizures persists for 14 years after stroke in this study (Supplementary Figure 1A). The two approved treatments for reperfusion following acute ischemic stroke are recombinant tissue plasminogen activator (r-tPA) administration and mechanical thrombectomy (31). A complication of reperfusion therapies in acute ischemic stroke is the risk of seizures (32). However, consistent with previous evidence, the correlation between thrombolytic therapy following ischemic stroke and PSE was still non-significant in this study (33–36). Ferreira-Atuesta C et al. revealed an interesting point that this association can involve treatment selection bias such as stroke severity and large-artery atherosclerotic etiology, which has a higher priority to receive reperfusion therapy (36). In addition, clinical symptoms indicating cerebral cortex injury, such as aphasia, visual field defects, and hemiparesis have also ever been reported as risk factors for PSE (14, 19, 21). We also found that aphasia was associated with PSE 1 and 5 years after ischemic insult. However, no significant association was observed for other focal neurological signs, such as hemiparesis or cranial nerve palsy symptoms.

Stroke severity is the most critical factor of outcomes in stroke patients. Our study found that stroke severity, as evaluated using the NIHSS score, length of hospitalization, and recurrence of stroke was strongly associated with PSE development. In our Cox regression model, moderate NIHSS scores (5–9) and severe NIHSS scores (≥10) were associated with 1.4-fold and 1.98-fold increases in the risk of PSE, respectively. In previous studies, a high NIHSS score at the time of stroke admission was consistently found to be a risk factor for PSE (9, 15, 17). In our study, the frequency of stroke recurrence was 18.9% during 16-year follow-up, similar to that in previous studies (6%−19%) (37–39), and stroke recurrence was independently associated with the development of PSE. Studies have also indicated that stroke recurrence results in an increase in PSE development (15, 40). Recurrent strokes tend to occur in the same vascular territory with the same pathophysiological mechanisms (41). Although the exact mechanism of the association between stroke recurrence and PSE has not yet been clarified, some investigations suggested that the accumulation of damages after strokes such as glial scar, reactive astrocytes, and reconstruction of the neural network are involved in the occurrence of PSE (42, 43) Similarly, length of hospital stay is also a factor reflecting the severity of stroke. The length of hospitalization for patients with PSE was higher than that for patients without PSE (44), consistent with our results.

Etiologies of ischemic stroke in young adults often include extracranial arterial dissection, cardioembolism, premature atherosclerosis, hematological and immunological disorders, migraine, heart diseases, pregnancy, and oral contraceptive use (45, 46). Nevertheless, large artery atherosclerosis, systemic vasculitis, cerebral aortic aneurysm, Moya Moya disease, and cardioembolism (atrial fibrillation, heart valve disease, coronary artery disease, myocardial infarction, cardiomyopathy, and heart failure) had no significant association with PSE development based on our analysis. These results are consistent with those of previous studies (18, 27). Moreover, the number of PSE patients with congenital metabolic, coagulopathic, and hematological diseases in this study was low for statistical analysis. Therefore, the association between these underlying conditions and PSE needs to be further investigated in future studies. Furthermore, malignancy is increasingly recognized as a risk factor for stroke in young adults (4). In this study, malignancy led to a 2-fold increase of PSE risk in Cox regression analysis. Although the toxic effects of chemotherapy and radiotherapy, hypercoagulable state, tumor embolism can explain stroke recurrence (47), the association between malignancy and PSE still needs to be studied further in the future.

Although hyperlipidemia is a risk factor for cerebrovascular disease (48), our findings revealed that hyperlipidemia was associated with a 34% decrease in the risk of PSE 1 year after stroke. Similarly, some studies also suggested that hyperlipidemia was more prevalent in the non-PSE group than in the PSE group (11, 49, 50). Yamada et al. reported that none single of the comorbidities, including hypertension, hyperlipidemia, diabetes, and coronary disease, conferred a significant correlation with PSE but the presence of more than one underlying disease was associated with a significantly lower probability of developing PSE (24). The lower incidence of PSE in stroke patients with dyslipidemia can be explained by the seizure-protective effects of lipid-lowering agents, statin used for this condition. Previous investigations have indicated that administering statins can decrease poststroke seizure occurrence and PSE (49, 51, 52). Therefore, we further evaluated the association between PSE and statin treatment in this study. Statins reduced the risk of PSE by 54% at 1 year and 44% at 5 years. The majority of patients diagnosed with hyperlipidemia were taking lipid-lowering agents which statins are the most common. This may explain why hyperlipidemia appeared as a protective factor for PSE in this study but it just reflected the effect of statin use. However, after controlling the statin treatment, we still observed an association between hyperlipidemia and PSE. We cannot find the related mechanism for supporting the protective effects of hyperlipidemia on PSE, but it still potentially exists beyond stain use. Certainly, more in-depth research is necessary to understand this phenomenon.

Unhealthy behaviors, such as drug abuse (3), smoking (25), and drinking (53), also had a significant impact on the increase in stroke rates among young people. Our results demonstrated that behaviors such as illicit and recreational drug use, smoking, and regular or heavy drinking are more common in PSE patients than that in non-PSE patients (*p* < 0.001, *p* = 0.003, and *p* = 0.012, respectively). Illicit and recreational drug use (cocaine, cannabis, and opioids) has been increasing over the past decade and is associated with stroke in the young population (29). Although the association between ischemic stroke and drug abuse has been reported in several studies (26, 29), none of them have demonstrated a correlation between illicit drug abuse and PSE in young adults. Our study is the first ones to show this association in the Cox regression model (HR, 2.9; 95% CI, 1.53–5.50). Some harmful effects of illegal drug abuse concerning ischemic stroke have been reported, such as chronic uncontrolled hypertension, vasospasm, enhanced platelet aggregation, cerebral vasculitis, accelerated atherosclerosis, and cardioembolism (54). These are all risk factors for stroke occurrence and recurrence. However, to explain the association between illicit drug abuse and PSE, the underlying mechanisms of this relationship need to be studied further in the future. In addition, evidence indicates that smoking is a well-established risk factor for ischemic stroke and stroke recurrence (5, 25, 55, 56). Stopping or even reducing the dose of smoking can also reduce the rate of stroke recurrence (56). Besides, heavy drinking can also significantly increase the risk of stroke because alcohol contributes to medical conditions that are risk factors for stroke, such as high blood pressure, diabetes, atrial fibrillation, and liver damage (53, 57). Recurrent stroke rate was elevated in patients with smoking and a history of alcohol abuse (58, 59). Although we observed a significant association between recurrent stroke and PSE in this study, our results failed to indicate that smoking and heavy drinking were independent risk factors for PSE. This is also consistent with some previous studies on the young population (9, 24, 27).

This study included a large cohort of young patients (*n* = 6512) which was highly representative of Taiwanese patients with ischemic stroke aged 19 through 44 years, and this study had a long follow-up duration of up to 16 years. Furthermore, multivariate regression models were used to reduce the influence of confounding factors. However, this study was retrospective, observational and therefore has some limitations. The NHIRD also lacks detailed clinical and laboratory information. In addition, the absence of code comparison with actual diagnoses may limit the reliability of results. This is also the general limitation of studies using a claim-based database.

## Conclusion

In summary, seizure at first admission, severity of stroke (high NIHSS score, prolonged hospital stay, and recurrent stroke), malignancy and drug abuse are associated increased risk of PSE in young population. Contradictory, use of statins is associated with decreased risk of PSE. This knowledge contributes to the provision of clinical evidence for prognostication, helping to identify populations that would benefit from preventive interventions reducing promptly epileptogenesis.

## Data Availability Statement

The original contributions presented in the study are included in the article/Supplementary Material, further inquiries can be directed to the corresponding authors.

## Ethics Statement

The studies involving human participants were reviewed and approved by Taipei Medical University Joint Institutional Review Board. Written informed consent for participation was not required for this study in accordance with the national legislation and the institutional requirements.

## Author Contributions

PTD, C-JH, and L-NC developed the original concept and design of the manuscript. L-NC, PTD, and C-JH performed the literature search and analysis of data in the literature, contributed drafted, reviewed, and edited the manuscript. All authors contributed to the article and approved the submitted version.

## Conflict of Interest

The authors declare that the research was conducted in the absence of any commercial or financial relationships that could be construed as a potential conflict of interest.

## Publisher's Note

All claims expressed in this article are solely those of the authors and do not necessarily represent those of their affiliated organizations, or those of the publisher, the editors and the reviewers. Any product that may be evaluated in this article, or claim that may be made by its manufacturer, is not guaranteed or endorsed by the publisher.
