# Supplementary material for: Risk Factors for Postischemic Stroke Epilepsy in Young Adults: A Nationwide Population-Based Study in Taiwan
Source: Front Neurol. 2022 May 20;13:880661. doi: 10.3389/fneur.2022.880661 (PMC9163822; doi:10.3389/fneur.2022.880661)
Supplement: Supplementary file 1 [file Table_1.DOCX]

Supplementary Material

# MATERIALS AND METHODS

Table 1. The Taiwan population-based claim data files in this study

| **Data file** | **Data name** |
| --- | --- |
| Health01 | Ambulatory care expenditures by visits |
| Health02 | Inpatient expenditures by admissions |
| Health03 | Expenditures for prescriptions dispensed at contracted pharmacies |
| Health04 | Details of ambulatory care orders |
| Health05 | Details of inpatient orders |
| Health06 | Details of prescriptions dispensed at contracted pharmacies |
| Health07 | Registry for beneficiaries |
| Health11 | Accreditation Profile of Medical Facilities |

Table 2. ICD-9 codes of comorbidities and behavioral risk factors in this study

| Diseases/lifestyles | ICD-9 code |
| --- | --- |
| Cerebral artery occlusion, large-artery atherosclerosis | 434, 440.* |
| Systemic vasculitis | 440.2–440.4, 443.9, 446, 446.29, 447.6, 437.4, 694.1, 710 |
| Cardioembolism | 745-746, 427.31, 427.9, 396.9, 397.9, 746.3, 996.02, 424.9, 410, 413–414, 429.2, 425.1, 425.4, 428 |
| Moya Moya | 437.5 |
| Ill-defined cerebrovascular disease, small vessel disease | 437.8 |
| Nervous system disease | 850–854, 959.01, 320, 321, 322, 323, 324, 326 |
| Congenital metabolic disorders | 277.87, 270.4 |
| Coagulopathy and hematology | 282.6, 289.81, 289.8, 286.0, 286.1, 286.9 |
| Hyperlipidemia | 272, 272.4 |
| Hypertension | 401-405 |
| Chronic head and neck disorders | 784 |
| Diabetes mellitus, type 2 | 250.0 |
| Migraine | 346 |
| Malignant neoplasm | 140–208 |
| Moderate or severe liver disease | 456–456.2, 572.2–572.4, 572.8 |
| Nephrotic syndrome | 581.9 |
| Renal disease | 403.01, 403.11, 403.91, 404.02–404.03, 404.12–404.13, 404.92, 404.93, 582, 583–583.2, 583.4, 583.6–583.7, 585–586, 588, V42, V45.1, V56 |
| Rheumatoid arthritis or collagen vascular disease | 446, 701, 710–710.4, 710.8–710.9, 711.2, 714, 719.3, 720, 725, 728.5, 728.89, 729.3 |
| Drug abuse | 292, 304, 305.2–305.9, V65.42 |
| Gravidity or postpartum period | V22.1, V22.2 |
| Tobacco smoking | 305 |
| Overweight/obesity | 278 |
| Regular drinking or recent heavy drinking | 303, 305 |

**FIGURE 1**. Cumulative risk of poststroke epilepsy. (A) Overall cumulative risk, and cumulative risk with (B), (C) stroke severity (NIHSS score, length of hospitalization), (D) drug abuse

1. **(B)**


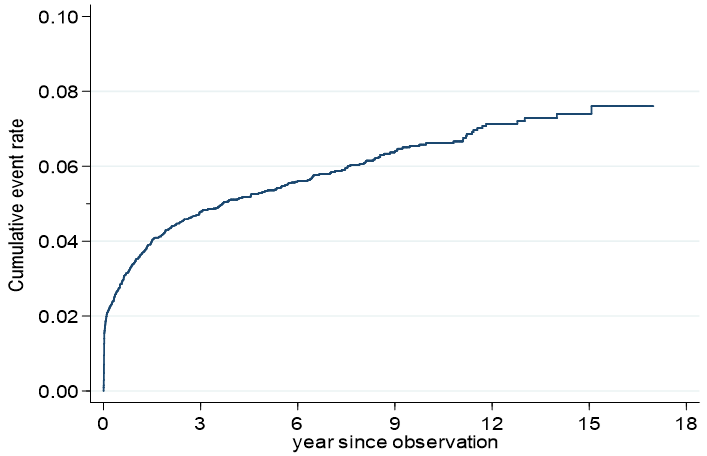

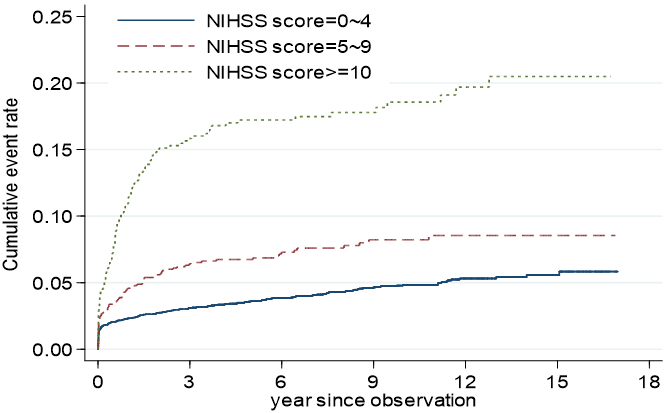


**(C) (D)**


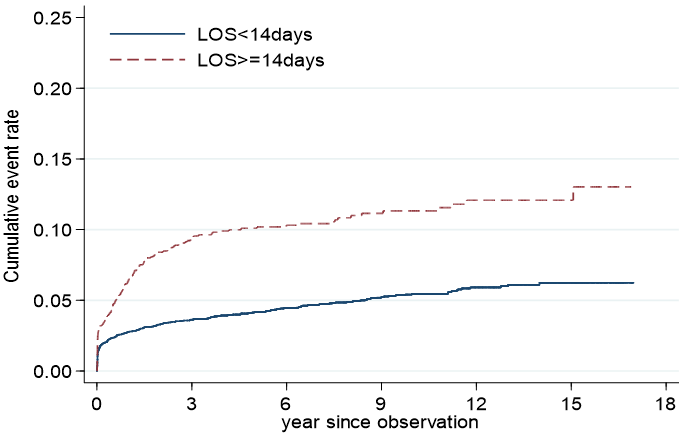

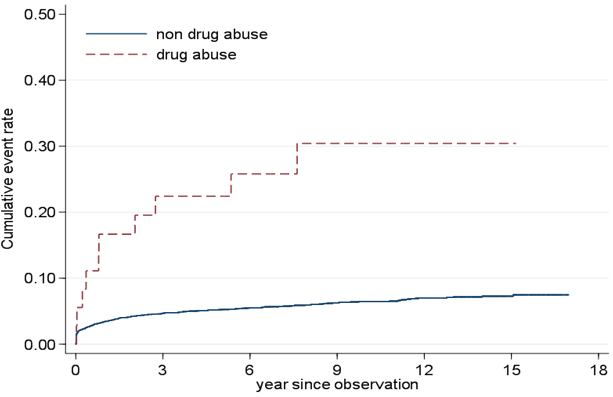


**FIGURE 2.** Association between hyperlipidemia and statin use with PSE. Cumulative PSE rate of (A) hyperlipidemia, and (B) statin use in hyperlipidemia patients

1. **(B)**


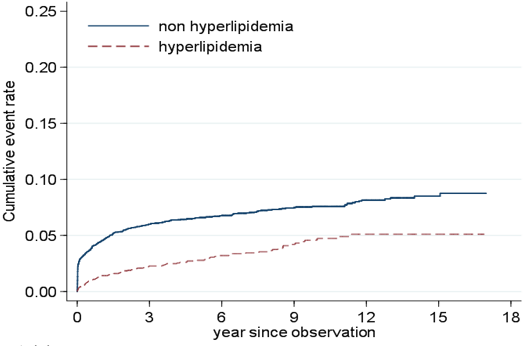

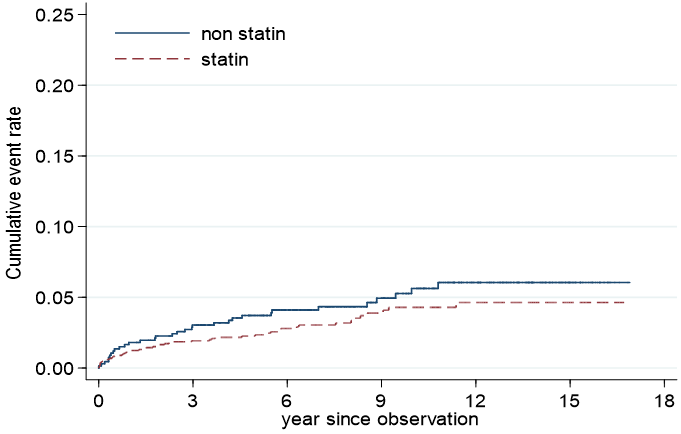


**
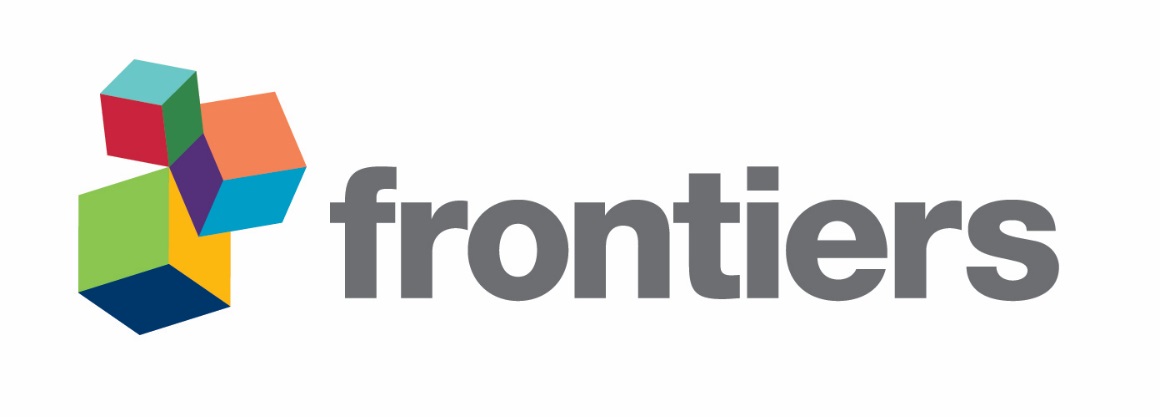
**

.
